# COVID-19 Presenting as Lupus Erythematosus-Like Syndrome

**DOI:** 10.1017/dmp.2020.358

**Published:** 2020-09-10

**Authors:** Sahar El Aoud, Clément Morin, Pauline Lorriaux, Julie Obert, Didier Sorial, Tarek Chaabouni, Laurent Thomas

**Affiliations:** Department of Internal Medicine, Saint Camille Hospital, Bry-Sur-Marne, France; Laboratory of Biology, Saint Camille Hospital, Bry-Sur-Marne, France

**Keywords:** acute kidney injury, COVID-19, lupus-like symptoms, multiorgan damages

## Abstract

The 2019 coronavirus disease (COVID-19) infection had newly emerged with predominant respiratory complications. Other extrapulmonary features had been recently described. Here, we describe a COVID-19 patient presenting with multiorgan involvement mimicking systemic lupus erythematosus. He was successfully treated with glucocorticoids and tocilizumab.

Coronavirus disease (COVID-19) uses angiotensin converting enzyme 2 as its functional receptor to infect endothelial target cells in lung, kidney, liver, heart, and the gastrointestinal tract. Therefore, in addition to respiratory symptoms that usually dominate the clinical presentation, there is recent increasing data showing that this novel coronavirus has potential extrapulmonary complications. Here, we report the case of the COVID-19 infection with multiorgan dysfunction, simulating systemic lupus erythematosus (SLE) presentation.

A 62-year-old man with no medical history was referred by his primary care physician to our Department of Internal Medicine on April 4, 2020, for shortness of breath associated with a kidney injury. He suffered from fever, cough, and myalgia 17 days before. On examination, the temperature was 39°C, the blood pressure 105/85 mm Hg, and the heart rate 97 beats per minute. His oxygen saturation was 94% on room air. He had bibasilar rales. A neuropsychiatric examination showed acute confusion, disorientation in time and space, and behavioral disorders. He had dysexecutive syndrome consisting of slowed thinking, information processing impairment, memory loss, inattention, and poorly and slowed organized movements in response to command.

Laboratory tests showed the white blood cell count at 10 900/mm^3^, lymphopenia at 800/mm^3^, 0 eosinophils per mm^3^, mildly increased liver enzymes, elevated C-reactive protein (CRP) concentration and procalcitonin at 208 mg/l and 2.41 ng/ml, respectively. Protein electrophoresis showed hypoalbuminemia at 19.7 g/l and elevated α 2 globulin at 11.8 g/l. He had a blood urea nitrogen at 7 µmol/L, blood creatinine at 204 µmol/L with an estimated glomerular filtration rate (EGFR) at 38 mL/min/1.73 m^2^. His baseline creatinine level was unknown. A urinalysis showed the protein-to-creatinine ratio at 233 mg/mmol with no evidence of hematuria, sodium 9 mmol/L, and potassium 36 mmol/L. Blood and urine cultures were negative. The laboratory results are shown in Table 1.

**TABLE 1:** Summary of Laboratory Results During Hospitalization

Hospital Day	1	4	5	10	11	14	17	19	22
Oxygen Therapy (L/min)	0	4	6	12	10	5	5	2	0
White-cell Count (per mm^3^)	10 900	10 400	8500	9800	6800	5400	4100	3800	2900
Total Neutrophils	9800	9000	7200	8400	5900	3500	1800	1200	1000
Total Lymphocytes	800	900	600	500	500	1100	1200	1100	1000
Total Eosinophils	0	0	0	100	0	100	200	300	200
Hemoglobin (g/dl)	13.9	13	12.9	9.9	11.3	10.6	10.6	10.1	10
Platelet Count (per mm^3^)	131 000	154 000	186 000	32 300	321 000	273 000	193 000	157 000	116 000
C-reactive Protein (mg/L)	208	250	159	277		39	10	4	2
Serum Ferritin (μg/L)				1500		1500	1416	1318	940
Procalcitonin (ng/ml)	2.41			2,86					
Urea (mmol/L)	7.01	18.37	20.88	10.52	11.69	9.69	6.35	5.01	2.84
Creatinine (μmol/L)	204	327	310	260	212	168	142	124	115
EGFR (ml/min/1.73 m^2^)	38	23	25	28	36	46	54	55	67
Albumin (g/L)	19.7								
D-Dimer (mg/L)				4380	2190	1190	930	740	480
Fibrinogen (g/L)				9.91		6.54	5.18	4.52	3.59
ASAT (U/L)	55			62	71	109	129	105	57
ALAT (U/L)	20			53	62	141	159	156	95
GGT (U/L)				275	262	453		329	268
ALP (U/L)	55			91	85		73	76	77
Triglyceride (g/L)				1.84	1.92				
LDH (U/L)				555	555	461		404	

*Notes*: EGFR, estimated glomerular filtration rate; ASAT, aspartate aminotransferase; ALAT, alanine aminotransferase; GGT, gamma-glutamyl transferase; ALP, alkaline phosphatase; LDH, lactate dehydrogenase.

Investigations for mycoplasma pneumoniae, human immunodeficiency virus, influenza A and B, hepatitis A, B, and C, and antineutrophil cytoplasmic antibodies were negative. Serum Complement 3 and 4 levels were normal. Antinuclear antibodies (ANA) were positive at 1/160. Anti-double stranded (ds) DNA and anti-extractable nuclear antigen antibodies were negative. A cerebrospinal fluid (CSF) examination and brain magnetic resonance imaging were normal. A lung computed tomography (CT) imaging revealed extensive lesions with ground-glass opacities, consolidations, and a crazy paving pattern with the extent of the lesions at 25% (Figure 1A). A CT scan of the abdomen and pelvis showed normal-sized kidneys with permeable arteries and urinary tract. In view of the pandemic context, reverse-transcriptase–polymerase-chain-reaction (RT-PCR) test for the virus responsible for COVID-19, severe acute respiratory syndrome coronavirus 2 (SARS-CoV-2), was performed. It was positive in the nasopharyngeal swab, nevertheless, and it was negative in CSF.

**FIGURE 1:**
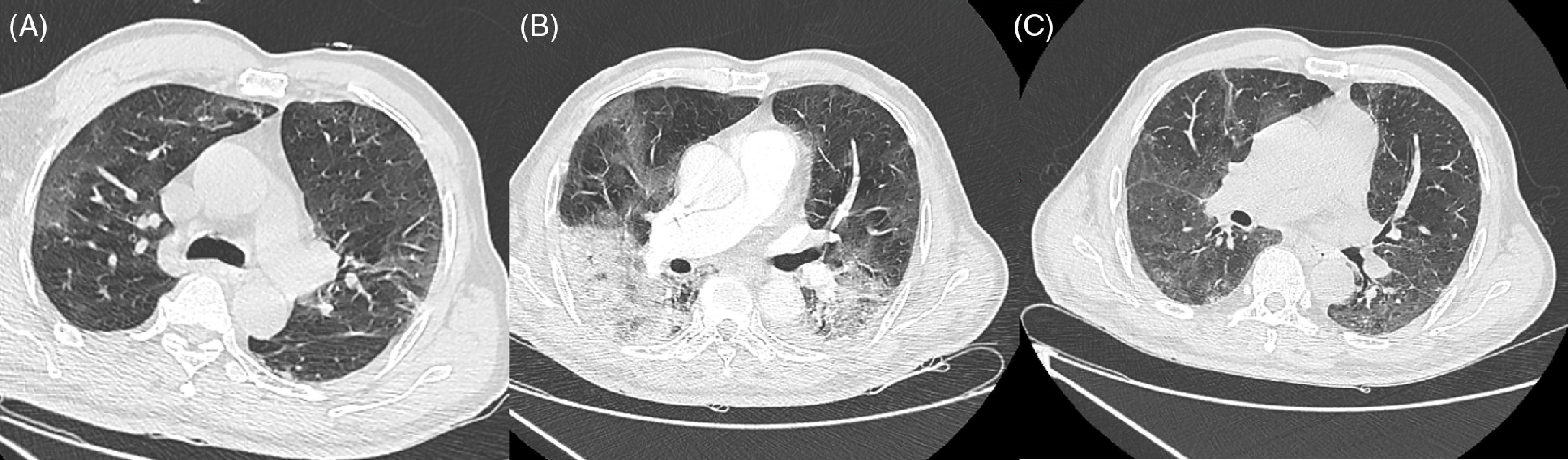
Lung CT Imaging Showing Pulmonary Lesions on Day 1 (A), Day 10 (B), and Day 52 (C).

The patient was started on empiric antibiotics (Rovamycine and ceftriaxone) and prophylactic anticoagulation. Three days after, the oxygen flow rate was progressively increased to 6 L/min. He had tachycardia at 120/min. His renal function worsened with his respiratory distress. Blood creatinine continued to rise to 325 µmol/L. His urine output remained between 1500 and 1800 ml/day. He was given curative anticoagulation due to pulmonary embolism suspicion. Angio CT of the thorax was not performed due to his renal function deterioration. He received progressive intravenous hydration with little improvement of his creatinine level that subsequently stabilized at 260 µmol/L.

On Day 10, the patient’s hypoxemia progressed requiring an oxygen flow at 12 L/min. He remained febrile at 39°C. A clinical examination showed cognitive and motor disorders worsening. Repeat kidney laboratory tests were notable for acute organic kidney injury (EGFR 28 ml/min/1.73 m^2^, urine sodium: 40 mmol/l, urine potassium: 29 mmol/l) with a protein-to-creatinine ratio at 200 mg/mmol, hematuria at 200/mm^3^, and leukocyturia at 100/mm^3^. Urine culture remained negative. An angio CT of the thorax, abdomen, and pelvis showed significant worsening of pulmonary lesions extent between 50 and 75% (see Figure 1B) with no evidence of embolism and splenomegaly. With a persistent fever, simultaneous declining of pulmonary, renal, and neurological status and biological inflammatory parameters rising, the treatment had been started with 120 mg of intravenous methylprednisolone for 2 repeated doses, tocilizumab (TCZ) at 600 mg, and Tazocilline. Two days later, corticoids were decreased to 80 mg for 2 days then 40 mg for 2 more days.

On Day 14, the oxygen requirement was progressively decreased, the neurological status was gradually improved, serum creatinine level decreased to 115 µmol/L, and inflammatory parameters regressed. After 22 days in the hospital, the patient was weaned from oxygen and transferred to a rehabilitation facility.

One month later, the patient had a normal neurological status. His serum creatinine level was stable at 120 µmol/L with EGFR at 62 ml/min/1.73 m^2^. His urinalysis showed the protein-to-creatinine ratio at 57 mg/mmol with neither hematuria nor leukocyturia. A lung CT scan revealed a significant improvement of his pulmonary lesions (see Figure 1C).

Lupus-like syndrome is usually induced by drugs, more rarely by infections activating autoimmune response by transient positive autoantibodies, which can be associated with lupus-like symptoms such as malar rash, arthralgia, nephritis, and acute hepatitis.^1^ Our patient’s clinical presentation, including glomerular syndrome with significant proteinuria and hematuria, neuropsychiatric symptoms, lymphopenia, and positive ANA, fulfilled the 2012 Systemic Lupus International Collaborating Clinics revised classification criteria. Hepatitis and interstitial pneumonia could be considered as supporting criteria for this diagnosis. Nevertheless, an anti-Sm (Smith antibody) and anti-dsDNA antibody test are more specific than ANA for lupus nephritis, which is usually associated with complement consumption. Therefore, the age and male gender of our patient, elevated CRP and procalcitonin, low ANA titer, negative anti-dsDNA and anti Sm, and normal serum complement level called into doubt the diagnosis of SLE. The diagnosis of COVID-19 with multiorgan damages was more probable.

Recently, some publications have illustrated the renal invasive potential of COVID-19. Notably, acute kidney injury (AKI) is noted in 9.2% of population in South Korea.^2^ In Cheng et al.’s large prospective cohort study including 701 patients with COVID-19,^3^ the prevalence of proteinuria, hematuria, EGFR under 60 ml/min/1.73m^2^, and AKI occurring during the study period was 43.9%, 26.7%, 13.1%, and 5.1%, respectively. A Chinese report on a renal histopathological analysis of 26 autopsies of patients with COVID-19 revealed acute proximal tubular injury, vacuolar degeneration, erythrocyte aggregation in peritubular and glomerular capillaries, glomerular fibrin thrombi, ischemic changes, and severe endothelial injury.^4^ COVID-19 particles were identified in the cytoplasm of renal proximal tubular epithelium, as well as in the podocytes with positive COVID-19 nucleoprotein in tubules by an indirect fluorescence method. Interestingly, rare cases of glomerulonephritis in patients with COVID-19 have been described.^5^

In addition to direct virus mediated injury, kidney involvement with a COVID-19 infection could be due to systemic hypoxia, hypercoagulability, microangiopathy, rhabdomyolysis, sepsis, cytokine storm, and angiotensin II pathway activation.^6^ Our patient had no cardiovascular factors risk. His volume contraction due to decreased oral input and insensible fluid loss associated with fever contributed to AKI, which explained little improvement of his renal function after fluid resuscitation. His urine dipstick test was compatible with glomerular syndrome and probable tubular disorders. Unfortunately, he did not have a renal biopsy because of safety concerns.

There is also an increasing evidence of central neurological complications of COVID-19, including headaches, acute stroke,^7^ encephalitis, and Guillain Barré syndrome. In Helms et al.’s report,^8^ including 58 patients hospitalized in the intensive care unit (ICU), confusion was noted in 26 of 40 patients and dysexecutive syndrome in 15 of 45 (33%), such as in the case of our patient.

Kidney and neurological complications were described in critically ill patients with COVID-19.^3,7^ Renal involvement is considered a new predictor of patient deterioration. It correlates with a poor prognosis and is associated with a higher risk of mortality.^3^

In recent reports, TCZ has been used in patients with COVID-19 and severe pulmonary involvement showing encouraging results with reduced ventilatory support requirements, especially in those with cytokine storm and higher IL-6 levels.^9,10^ The most used medications in COVID-19 patients with renal complications are diuretics, glucocorticoids, antivirus, and renal replacement therapy.^3^ To our knowledge, preliminary data on TCZ use in kidney complications are very limited. Interestingly, our patient was successfully treated with glucocorticoids and TCZ, preventing admission in ICU with significant improvement of kidney, respiratory, neurological, and liver damages.

COVID-19 can induce kidney, neurological, liver, and vascular damage with possible positive ANA simulating multisystem inflammatory disease. Renal dysfunction is rare during this infection and usually associated with a fatal clinical course. Fortunately, our patient was discharged from the hospital with favorable outcome after glucocorticoids and TCZ treatment. Therefore, regulatory monitoring of inflammatory parameters, creatinine, and urine sample analysis allows an early disease worsening prediction leading to appropriate therapeutic options onset in critically ill patients.
